# Total flavonoids of *Dracocephalum moldavica* L. alleviate cognitive impairment via TNF-α/NF-κB p65 signaling pathway in vascular dementia rats

**DOI:** 10.3389/fphar.2025.1640272

**Published:** 2025-08-06

**Authors:** Shangjia Ma, Jianxin Jia, Lie Wu, Kai Tian, Lu Wang, Hua Li, Jiayu Lv, Dewang Gao, Zhanjun Yang, Xia Guo

**Affiliations:** ^1^ Department of Neurology, The First Affiliated Hospital of Baotou Medical College, Baotou, China; ^2^ Department of Human Anatomy, Baotou Medical College, Baotou, China; ^3^ Key Laboratory of Human Anatomy, Education Department of Inner Mongolia Autonomous Region, Baotou, China; ^4^ Department of Psychological Rehabilitation, The Third Hospital of Baogang Group, Baotou, China; ^5^ Department of Human Anatomy, Chifeng University, Chifeng, China

**Keywords:** total flavonoids of *Dracocephalum moldavica* L., vascular dementia, network pharmacology, neuroinflammation, TNF-α/NF-κB p65 signaling pathway, blood-brain barrier

## Abstract

**Objective:**

This study aims to elucidate the neuroprotective effects and underlying mechanisms of total flavonoids of *Dracocephalum moldavica L.* (TFDM) in VaD by using network pharmacology and *in vivo* validation.

**Methods:**

The network pharmacology was used to explore the mechanism of TFDM to improve VaD. A rat model of VaD was established using permanent bilateral common carotid artery occlusion (2VO). The Morris water maze test assessed spatial learning and memory capacities. Nissl staining was used to examine the neuronal damage. Western blot and Immunofluorescence analysis was employed to evaluate protein levels of factors associated with neuroinflammation, blood-brain barrier integrity, and angiogenesis.

**Results:**

The network pharmacology suggests TFDM may combat VaD through TNF-α/NF-κB p65 signaling pathways. TFDM treatment may attenuate memory deficits associated with 2VO and reduce neuronal damage. TFDM improved blood-brain barrier integrity and promoted angiogenesis by downregulating MMP-9 and upregulating ZO-1 and VEGFA. Moreover, TFDM exhibited anti-inflammatory properties by inhibiting TNF-α and NF-κB p65 production, thereby mitigating the neuroinflammatory response in VaD rats.

**Conclusion:**

TFDM demonstrated significant improvement in cognitive function in VaD rats. This improvement was attributed to the multifaceted effects, including the improvement of blood-brain barrier integrity, promotion of angiogenesis, and reduction of neuroinflammation. These findings suggest that TFDM may represent a promising therapeutic approach for VaD management.

## 1 Introduction

Vascular dementia (VaD), the second most prevalent form of dementia, accounts for approximately 20% of all dementia cases (Morgan and Mc Auley, 2024). This degenerative disorder, characterized by impaired cognition and memory, is precipitated by various cerebrovascular conditions (O'Brien and Thomas, 2015). Patients with VaD often suffer from a diminished quality of life and impose significant financial and medical burdens on both families and society (Sontheimer et al., 2021). Currently, VaD treatment primarily involves non-cholinergic medications such as memantine, nimodipine, and hydergine, as well as cholinesterase inhibitors, including donepezil, rivastigmine, and galantamine (Xiao et al., 2024). However, these therapeutic agents exhibit limited clinical efficacy and notable side effects, mainly due to the poorly understood pathogenic mechanisms underlying VaD (Battle et al., 2021). Consequently, elucidating the disease mechanisms and identifying potential targets and therapeutic interventions for VaD is of paramount importance.

VaD has been associated with multiple pathological processes and potential underlying mechanisms. These include cerebral hypoperfusion, oxidative stress, trophic uncoupling, neuronal death, neuroinflammation, white matter lesions, and increased blood-brain barrier (BBB) permeability (Hosoki et al., 2023). However, the precise etiology of VaD remains elusive due to its complex nature. Neuroinflammation, characterized by activated microglia cells, overexpressed pro-inflammatory cytokines, and overactivation and infiltration of immune cells in the brain, contributes to numerous detrimental effects in VaD patients (Poh et al., 2022). Accumulating evidence suggests that neuroinflammation leads to neuronal cell death, tissue matrix degradation, and blood-brain barrier dysfunction (Tian et al., 2022). The BBB, a highly selective permeability barrier, is primarily composed of specialized endothelial cells incorporating complex tight junction proteins such as claudin-5, occludin, and zonula occludens-1 (ZO-1), along with astrocyte end-feet and pericytes (Greene et al., 2020; Otani and Furuse, 2020). Matrix metallopeptidases (MMPs) have been shown to increase permeability, compromise BBB tight junction integrity, and ultimately result in irreversible BBB damage (Candelario-Jalil et al., 2022). Studies have demonstrated that suppression of neuroinflammation and protection of BBB permeability improve cognitive disorders in rat models of VaD (Thangwong et al., 2023; Zhou et al., 2023). Therefore, strategies targeting anti-neuroinflammation and BBB improvement could prove beneficial for maintaining nervous system function and ameliorating cognitive dysfunction in VaD patients.

*Dracocephalum moldavica* L., a traditional Chinese herbal remedy derived from a member of the Labiatae family, is abundant in flavonoids, terpenoids, and phenylpropanoid chemicals. The total flavonoids of *Dracocephalum moldavica* L. (TFDM) have been reported to exhibit hepatoprotective and cardioprotective properties, ameliorate pulmonary fibrosis, prevent cognitive decline, and possess detoxifying effects on the spleen and liver (He et al., 2023; Kim et al., 2021; Zhan et al., 2024). In middle cerebral artery occlusion model mice, TFDM has shown neuroprotective benefits that may be related to suppressing the release of inflammatory mediators linked to apoptosis (Wu et al., 2018). Furthermore, TFDM has been shown to reduce the production of inflammatory cytokines and neurotoxicity in Alzheimer’s disease models (Ren et al., 2024).

Network pharmacology has emerged as a potent tool for investigating complex drug compounds and their functions due to the quick development of bioinformatics technology (Wang et al., 2024). It is frequently employed in identifying possible therapeutic ingredients in traditional Chinese medicine and the molecular-level prediction of their potential pharmacological mechanism targets (Nogales et al., 2022). First, we hypothesized that TFDM, possibly through the Tumor necrosis factor-alpha (TNF-α)/Nuclear factor kappa B p65 (NF-κB p65) signaling pathway, would minimize neuroinflammation and control cognitive impairment following VaD. Experiments are then used to confirm the mechanism as mentioned above. By utilizing 2VO model rats, we investigated whether TFDM treatment could alleviate cognitive impairment by attenuating neuroinflammation and enhancing angiogenesis and blood-brain barrier integrity. By leveraging the advantages of traditional Chinese herbs, this research provides a more profound, comprehensive understanding of TFDM as a potential therapeutic agent for VaD.

## 2 Materials and methods

### 2.1 Materials

The antibodies utilized in this study, including NF-κB p65, Vascular endothelial growth factor A (VEGFA), ZO-1, β-actin, and secondary antibodies, were procured from Affinity Company, United States. TNF-α and Matrix metallopeptidases-9 (MMP-9) antibodies were obtained from Proteintech Company, United States. TFDM was distilled and acquired from PuYi Biotech, Nanjing, China. Donepezil hydrochloride (DPH) was sourced from WeiCai Pharmaceutical Co., Ltd., China. Both TFDM and donepezil hydrochloride were solubilized in distilled water prior to use.

### 2.2 Network pharmacology

#### 2.2.1 Identifying components and targets of TFDM and VaD

The PubChem database, Literature mining, and the 2020 edition of the Chinese Pharmacopoeia were employed to search and collect the components and targets of TFDM. Subsequently, The TFDM active ingredients and Simplified Molecular Input LineEntry System (SMILES)codes were acquired and imported into the Swiss Target Prediction database to predict their targets. The keyword “vascular dementia” was searched and screened on the Gene Cards database and OMIM database to obtain targets. The VENNY website was used to acquire the co-targets of VaD and TFDM.

#### 2.2.2 Constructing the “component-target-disease” visualization network

The co-targets of VaD and TFDM were imported into Cytoscape 3.9.2 software for network topology analysis to construct a component-target-disease visualization network.

#### 2.2.3 Constructing the protein-protein interactions network

The co-targets of VaD and TFDM were imported into the String website to structure the PPI network. *Homo Sapiens* was a limited Organism, and the minimum required interaction score was set to the highest confidence (0.900). Finally, the PPI network diagram was drawn using Cytoscape 3.9.2 software, which used the top 10 Degree values as core targets.

#### 2.2.4 GO enrichment analysis

The co-targets of VaD and TFDM were imported into the clusterProfiler, and Stringin softwares for the GO analysis (*P* ≤ 0.01) to clarify the target function of active components in TFDM. The top 10 entries were then chosen individually to be shown using the Microbiology Platform.

#### 2.2.5 KEGG analysis

The clusterProfiler, and Stringin softwares performed a KEGG analysis of important targets to investigate the precise pathways involved in the effect of TFDM on VaD. The top 10 paths that had a cut-off value of *P* < 0.05 were kept.

### 2.3 Experimental animals

The 60 male, healthy Wistar rats (6–8 weeks old, weighing 230–270 g) were procured from SPF (Beijing) Biotechnology Co., Ltd. [License No. SCXK (Jin) 2019–0010]. The rats were maintained in the Animal Experiment Center of Baotou Medical College. The animals were provided *ad libitum* access to food and water. They were housed under controlled environmental conditions, including a 12-hour light/dark cycle, an ambient temperature of 23°C ± 1°C, and a 50% humidity level. The experimental protocol was approved by the Baotou Medical College Animal Ethics Review Committee [2023] No. 47 for ethics review.

### 2.4 Permanent bilateral common carotid artery occlusion (2VO) rat model

**FIGURE 1:**
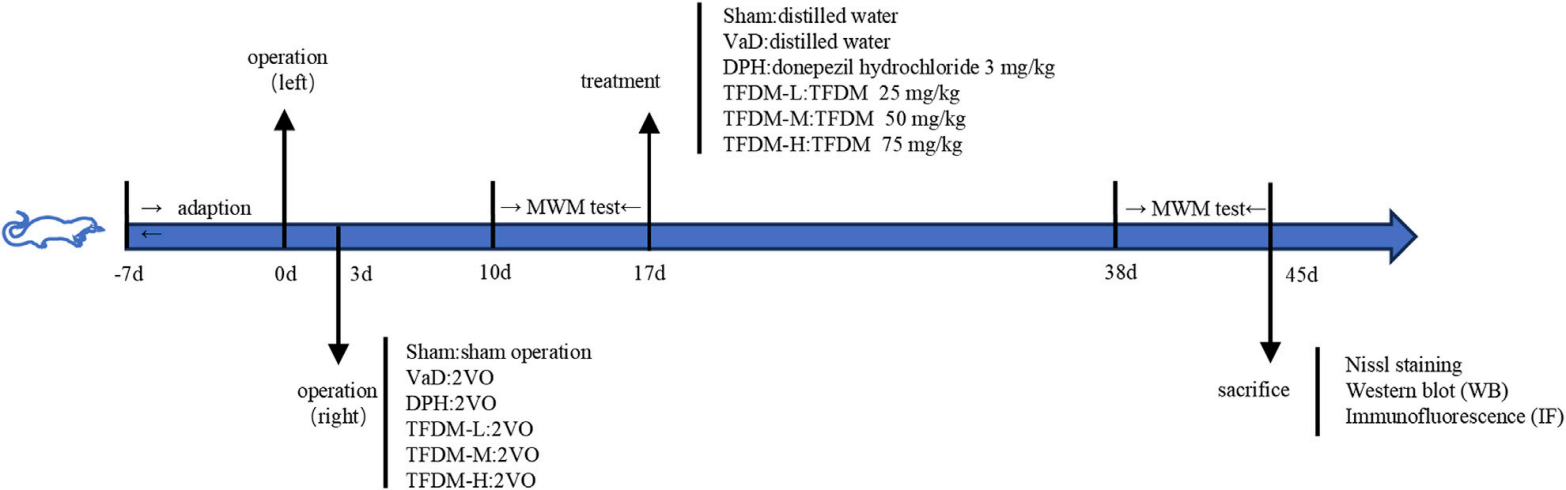
A complete flow chart of this study.

The 2VO model was established using the internationally recognized modified permanent bilateral common carotid artery occlusion procedure. The rats were acclimatized and fed for 1 week, and 50 of them were operated upon by 2VO to successfully establish a VaD model, while the remaining 10 served as the Sham group with bilateral carotid artery exposure only, without ligation. Anesthesia was induced in rats using 2% sodium pentobarbital. The left common carotid artery was exposed and ligated using a surgical thread. After a 3-day interval, the same procedure was performed on the right common carotid artery. 1 week after operation, the Morris Water Maze test was employed to verify the successful establishment of the 2VO model. Successfully modeled rats were randomly assigned to five groups: VaD, DPH (3 mg/kg), TFDM-L (TFDM low dose, 25 mg/kg), TFDM-M (TFDM medium dose, 50 mg/kg), and TFDM-H (TFDM high dose, 75 mg/kg) (n = 10/group). Following a 1-day acclimatization, rats in the treatment groups received daily oral gavage treatment for 21 consecutive days with the corresponding drug dosages. The VaD group and the sham group were administered an equivalent volume of distilled water. A comprehensive flow chart of this study is presented in Figure 1.

### 2.5 Morris water maze (MWM) test

The MWM test consisted of a circular water-filled tank divided into four quadrants. An escape platform was submerged approximately 2 cm below the water surface (22°C ± 1°C) in the fourth quadrant. During the initial 5 days of training trials, rats were sequentially released from each quadrant into the water, facing the pool wall. The total swimming distance and escape latency time were recorded and analyzed for 60 s. If a rat failed to locate the platform within 60 s, it was guided to adapt for 10 s, and its escape latency was recorded as 60 s. On the sixth day, a probe trial was conducted with the platform removed. The percentage of time stayed in the target quadrant, and the number of platform location crossings was measured over 120 s to assess spatial memory function. (n = 8/group).

### 2.6 Nissl staining

All rats were anesthetized with 2% pentobarbital sodium and perfused with 0.9% saline, followed by 4% paraformaldehyde via the ascending aorta and left cardiac ventricle. The brains were immediately extracted, fixed in 4% paraformaldehyde for 48 h, and embedded in paraffin. Brain sections underwent a 40-minute staining process using toluidine blue, followed by washing in ultrapure water and 95% ethanol. The sections were then cleared with xylene and sealed with neutral glue. The number of neurons in the cortex and CA1 region of the hippocampus was quantified using ImageJ software (n = 4/group).

### 2.7 Western blot

The hippocampus tissue was homogenized in RIPA Lysis Buffer and centrifuged for 5 min at 12,000 rpm. The hippocampus tissue protein was extracted and quantified using a BCA protein detection kit. Samples were denatured for 15 min at 100°C after being prepared with loading buffer. Following electrophoresis and membrane transfer, primary antibodies (1:1,000) against NF-κB p65, VEGFA, ZO-1, TNF-α, and MMP-9 were incubated overnight at 4°C. After washing with TBST, secondary antibodies (1:5,000) were applied for 2 h. The PVDF membrane was coated with ECL luminescent reagent, and images were captured using an automatic gel imaging analysis system (n = 5/group).

### 2.8 Immunofluorescence

Sections of paraffin were first blocked, permeabilized, and dewaxed. After adding NF-κB p65, VEGFA, ZO-1, and TNF-α antibodies (1:100), the mixture was incubated in a moist box at 4°C for the entire night. After adding diluted secondary antibodies (1:500), the mixture was incubated in a moist box at 37°C for 1 hour. NF-κB p65, VEGFA, ZO-1, and TNF-α expression were measured, and images were taken using a fluorescence-inverted microscope following a 5-minute incubation with DAPI at room temperature and three 5-minute PBS rinses (n = 3/group).

### 2.9 Statistical analysis

Statistical analyses were performed using GraphPad Prism 9.4 and SPSS 26.0 software. Optical density in Western blot and Immunofluorescence experiments was measured using ImageJ software. A one-way analysis of variance (ANOVA) followed by least significant difference (LSD) post-hoc test was employed to compare data between groups. Morris water maze test data were analyzed using a repeated-measures two-way ANOVA followed by LSD post-hoc test. Results are presented as mean ± standard deviation (SD), with statistical significance set at *P* < 0.05.

## 3 Results

### 3.1 Network pharmacology

#### 3.1.1 Identification of effective compounds of TFDM and target prediction of TFDM and VaD

**FIGURE 2:**
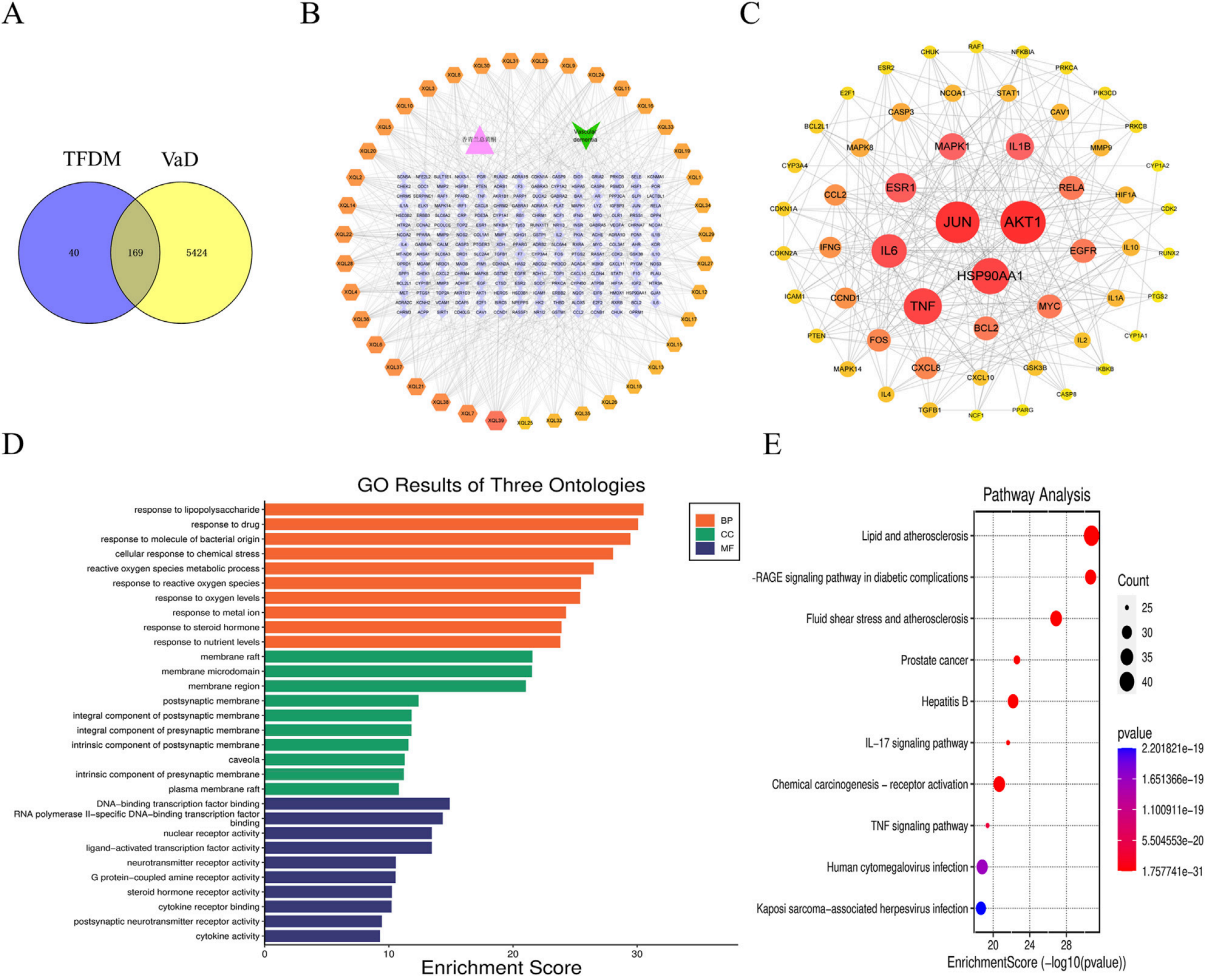
Network pharmacology-based strategy for predicting the possible mechanisms in TFDM for treating VaD. **(A)** The Venn diagram shows the targets in both TFDM and VaD. **(B)**” TFDM-target-VaD” network analysis. **(C)** The PPI network. **(D)** GO enrichment analysis. **(E)** and KEGG enrichment analysis.

Using the PubChem database, Literature mining, and the 2020 edition of the Chinese Pharmacopoeia, 39 active ingredients of TFDM were identified. 209 target genes of TFDM were gathered. In addition, 5,593 target genes of VaD were gathered. Venny software was applied to obtain 169 co-targets as potential key targets for TFDM for VaD (Figure 2A).

#### 3.1.2 “TFDM-target-VaD”network analysis and PPI network

The co-targets were imported into Cytoscape 3.9.2 software to construct a “TFDM-target-VaD” network diagram, which showed that 39 active ingredients of TFDM could potentially be used to treat VaD through these 169 targets (Figure 2B). Subsequently, the PPI network was analyzed by the String database, limiting the species to “*Homo sapiens*” with a score >0.90, and the discrete protein sites were proposed to obtain the PPI network (Figure 2C). Among these, the nodes possessing the top 10 are as follows: AKT1, JUN, TNF, HSP90AA1, IL-6, ESR1, 1L-1β, MAPK1, RELA, EGFR (Figure 2C; Table 1). Consequently, they were predicted to be the critical targets for DSS in the therapy of VaD.

**TABLE 1:** The core targets List.

No.	Target	BetweennessCentrality	ClosenessCentrality	Degree
1	AKT1	0.130035391	0.434640523	30
2	JUN	0.238350725	0.458620690	29
3	TNF	0.103617508	0.439558360	25
4	HSP90AA1	0.107205214	0.387755102	25
5	IL-6	0.080922535	0.413043478	23
6	ESR1	0.044113877	0.403030303	20
7	1L-1β	0.027221147	0.386627907	18
8	MAPK1	0.038222429	0.407954600	18
9	RELA	0.36372511	0.392330383	16
10	EGFR	0.59446964	0.390029326	16

#### 3.1.3 GO enrichment analysis and KEGG enrichment analysis

The Microbiology Platform, clusterProfiler, and Stringin softwares were used to perform GO enrichment and KEGG pathway analysis of the targets in the PPI network. The top 10 terms of BP, MF, and CC with the lowest P value were presented in Figure 2D. The significant terms of BP included response to lipopolysaccharide and reactive oxygen species metabolic process, response to reactive oxygen species, et al. The significant terms of CC included membrane raft, membrane microdomain, and membrane region. The significant terms of MF included DNA−binding transcription factor binding, nuclear receptor activity, et al. In addition, 257 KEGG pathways were recognized after KEGG pathway analysis, and the top 10 KEGG pathways with significantly adjusted P-values were presented in Figure 2E. The significant terms of signaling pathways IL−17 signaling pathway,TNF signaling pathway,et al.

### 3.2 TFDM improves the learning and cognitive function of VaD rats

**FIGURE 3:**
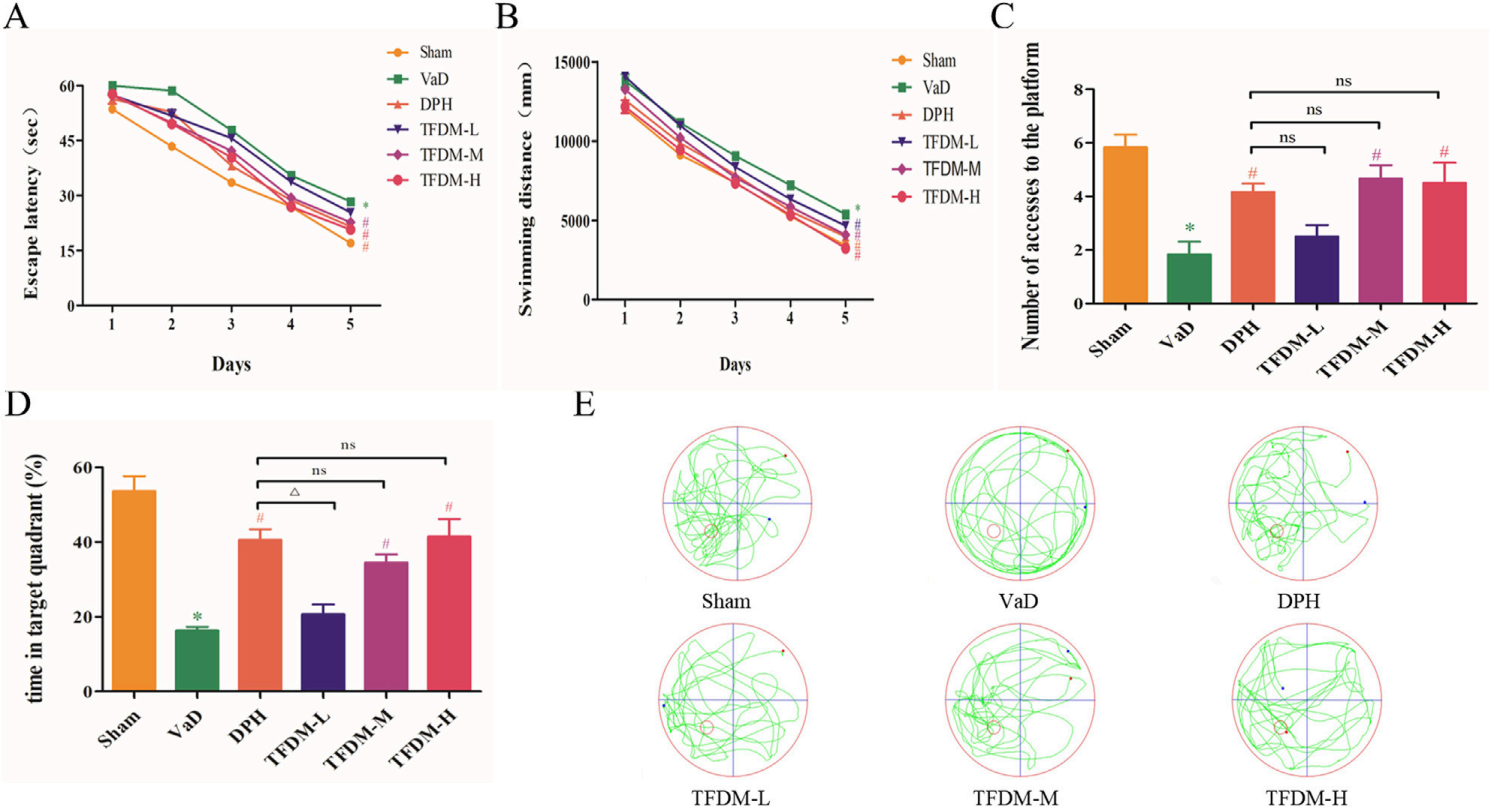
TFDM ameliorated cognitive impairment in VaD rats. **(A)** The effects of TFDM on the escape latency **(B)**, swimming distance **(C)**, number of accesses to the platform **(D)**, and time in the target quadrant (%) of VaD rats in the MWM experiment. **(E)** Typical path map of spatial exploration in VaD rats in the MWM experiment. The data are expressed as the mean ± SD (n = 8). ***P < 0.001, compared with the sham group. ###*P* < 0.001, ##*P* < 0.01, compared with the VaD group. △△*P* < 0.01, compared with the DPH group.

The MWM test was employed to evaluate the learning and memory retention abilities of six groups of rats. The findings revealed that in comparison to the sham group, rats in the VaD group exhibited significantly longer swimming distances and escape latencies during the training phase (*P* < 0.05 or *P* < 0.001). This observation may be indicative of decreased learning capacity in VaD rats. However, the escape latency time and swimming distance were reduced to varying degrees in the DPH, TFDM-M, and TFDM-H groups compared to the VaD group, suggesting that these treatments effectively prevented learning deficits in VaD rats (Figures 3A,B). Concurrently, the spatial memory capacity was assessed after platform removal. The number of accesses to the platform and time in target quadrant of rats in the VaD group were significantly lower than those in the sham group, indicating a decline in spatial memory in VaD rats (*P* < 0.001). Notably, the number of accesses to the platform and time in target quadrant were efficiently increased to varying degrees following DPH, TFDM-M, and TFDM-H dose treatments compared to the VaD group (Figures 3C,D). However, no significant differences were observed between these treatment groups. The motion trajectory figure illustrates the aimless search strategy employed by the VaD rats. In contrast, the sham, DPH, and all TFDM dosage rats utilized a goal-based search strategy (Figure 3E). These findings strongly suggest that TFDM treatment could effectively reverse the memory and learning deficits observed in VaD rats.

### 3.3 TFDM mitigated neuronal damage in VaD rats

**FIGURE 4:**
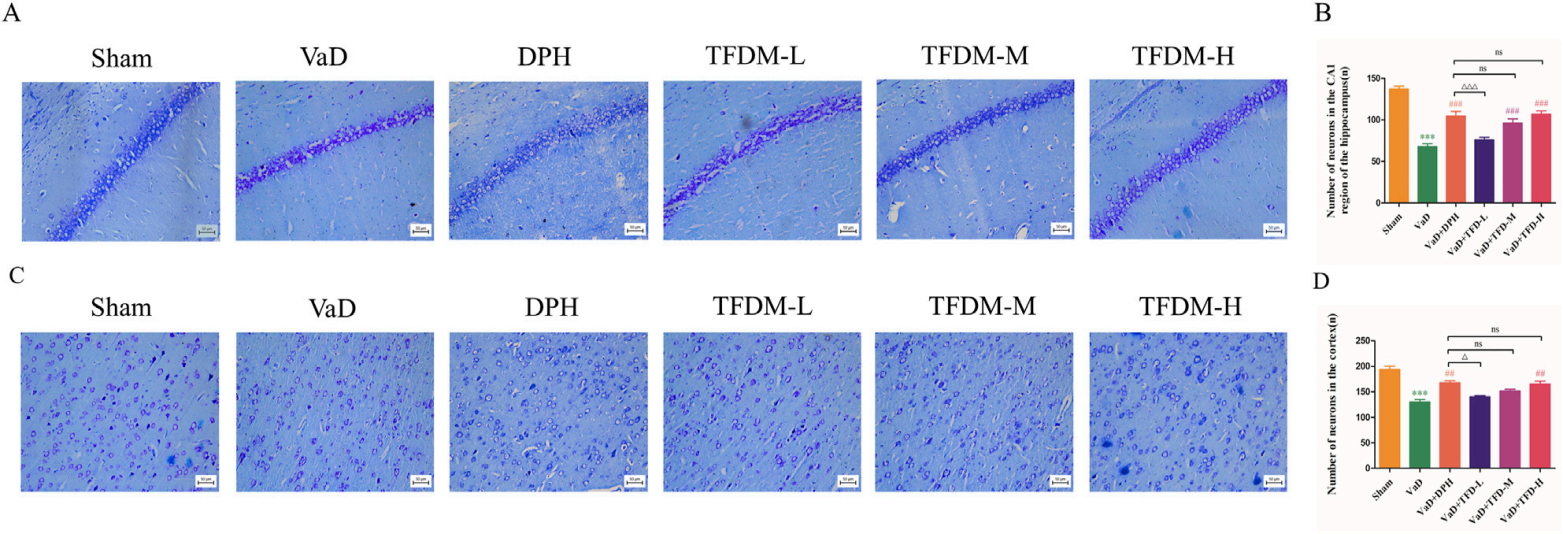
TFDM ameliorated neuronal damage in VaD rats. **(A,B)** Representative images and quantitative analysis of Nissl staining in the hippocampal CA1 region of rats (25um). **(C,D)** Representative images and quantitative analysis of Nissl staining in the rat cortex (25um). Data are presented as mean ± SD (n = 4). ***P < 0.001, compared with the sham group. ###*P* < 0.001, ##*P* < 0.01, compared with the VaD group. ΔΔΔ*P* < 0.001, Δ*P* < 0.05, compared with the DPH group. Data were analyzed by one-way ANOVA.

As illustrated in Figures 4A,C, Nissl staining was used to evaluate the histological structure of the hippocampus CA1 region and cortex. The results demonstrated that neurons in the sham group were fully formed and systematically arranged, with visible nucleoli and abundant, uniformly distributed Nissl bodies in the cytoplasm. In contrast, neurons in the VaD group exhibited structural damage and cellular disorganization. A large number of apoptotic cells were observed, and Nissl bodies in the cytoplasm had disintegrated and disappeared. Following the administration of DPH and all TFDM doses, the arrangement of neuron cells became more orderly and uniform, with rare occurrences of apoptotic and pyretic cells. A considerable increase in Nissl bodies was also noted. Statistical analysis (Figures 4B,D) revealed significant differences in the number of neuron cells among the groups in both the hippocampal CA1 region and cortex of the rats. The number of neurons in the VaD group was significantly lower than that of the sham group (*P* < 0.001). Compared to the VaD group, the number of neuron cells in the DPH, TFDM-M, and TFDM-H groups increased significantly after administration (*P* < 0.05 or *P* < 0.001). However, no significant differences were observed between these treatment groups. These findings suggest that TFDM treatment could attenuate neuronal damage in VaD rats.

### 3.4 TFDM protected blood-brain barrier permeability in VaD rats

**FIGURE 5:**
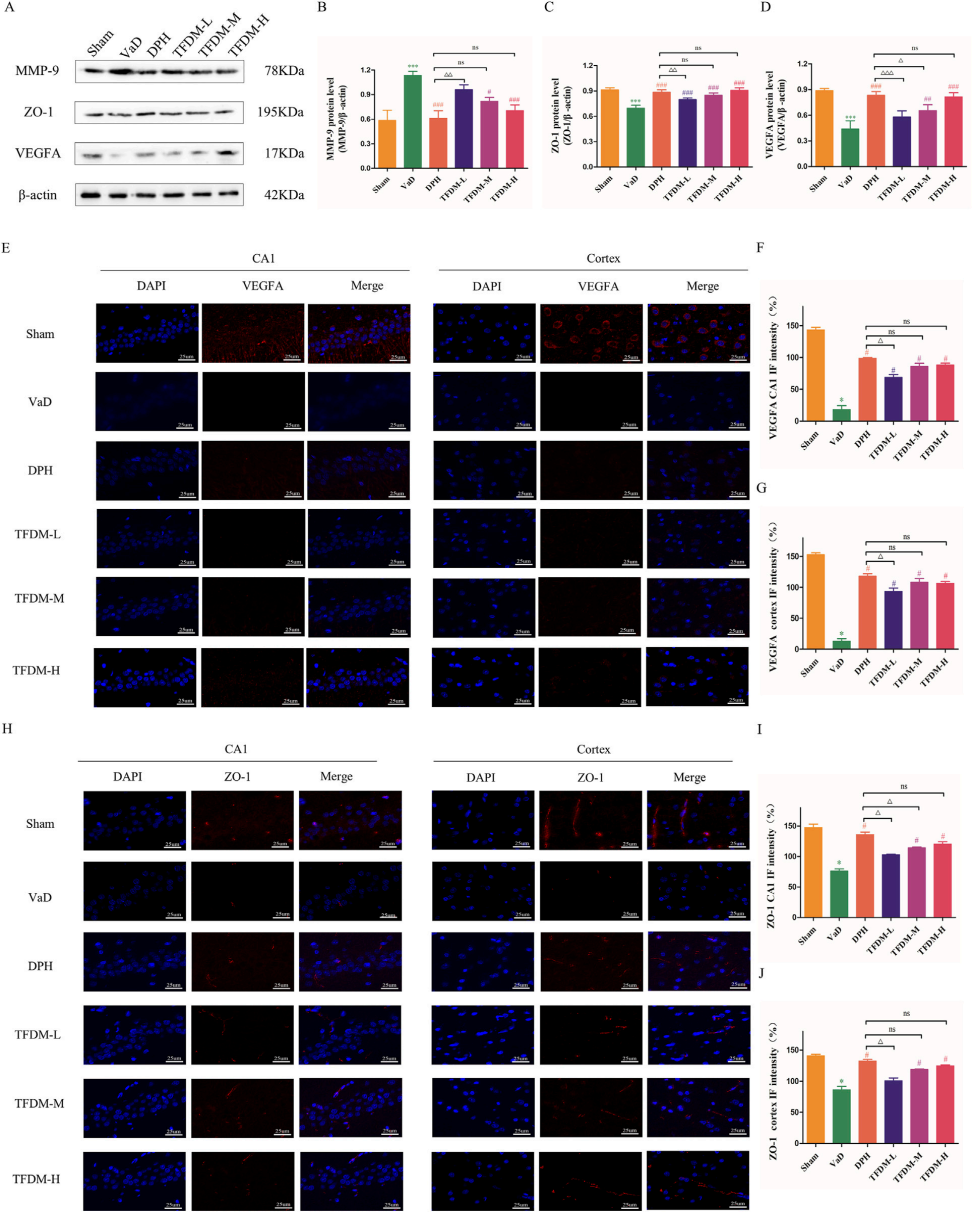
Effects of TFDM treatment on hippocampal BBB damage and angiogenesis. **(A)** Representative image of the Western blotting assay evaluating ZO-1, MMP-9, and VEGFA protein levels in rat brain tissue from each group. **(B–D)** Quantification results of Western blotting assays. **(E–G)** The immunofluorescence expression of VEGFA (red) in the cortex and CA1 region of the hippocampus (25um). **(H–J)** The immunofluorescence expression of ZO-1 (red) in the cortex and CA1 region of the hippocampus (25um). Data are expressed as mean ± SD (n = 5). The 100% reference represents the Sham group.***P < 0.001, compared with the sham group. ###*P* < 0.001, ##*P* < 0.01, #*P* < 0.05, compared with the VaD group. ΔΔΔ*P* < 0.001, ΔΔ*P* < 0.01, Δ*P* < 0.05, compared with the DPH group.

As illustrated in Figure 5A, Western blot analysis was employed to assess the proteins levels associated with ZO-1, MMP-9, and VEGFA to elucidate the regulatory effects on the blood-brain barrier and angiogenesis of TFDM. The results demonstrated a significant decrease in VEGFA and ZO-1 protein levels in the VaD group (*P* < 0.001) (Figures 5B,D), accompanied by an increase in MMP-9 protein levels compared to the Sham group (*P* < 0.001) (Figure 5C). Following drug intervention, VEGFA and ZO-1 protein levels was elevated, while MMP-9 protein levels was reduced in the DPH, TFDM-M, and TFDM-H groups compared to the VaD group (*P* < 0.05 or *P* < 0.001). Meanwhile, Immunofluorescence staining showed that the fluorescence intensity of VEGFA and ZO-1 in the cortex and hippocampal CA1 region was decreased in the VaD group compared to the Sham group (*P* < 0.001) (Figures 5E–J). However, the fluorescence intensity of VEGFA and ZO-1 in the cortex and hippocampal CA1 region was increased in TFDM-M, TFDM-H groups, and DPH group compared to the VaD group (*P* < 0.001 or *P* < 0.01 or *P* < 0.05) (Figures 5E–J). However, no significant differences were observed between these treatment groups. These findings suggest that TFDM therapy may alleviate cognitive impairment in VaD rats by protecting BBB permeability and promoting neovascularization.

### 3.5 TFDM suppressed the TNF-α/NF-κB p65 signaling pathway and neuroinflammation in VaD rats

**FIGURE 6:**
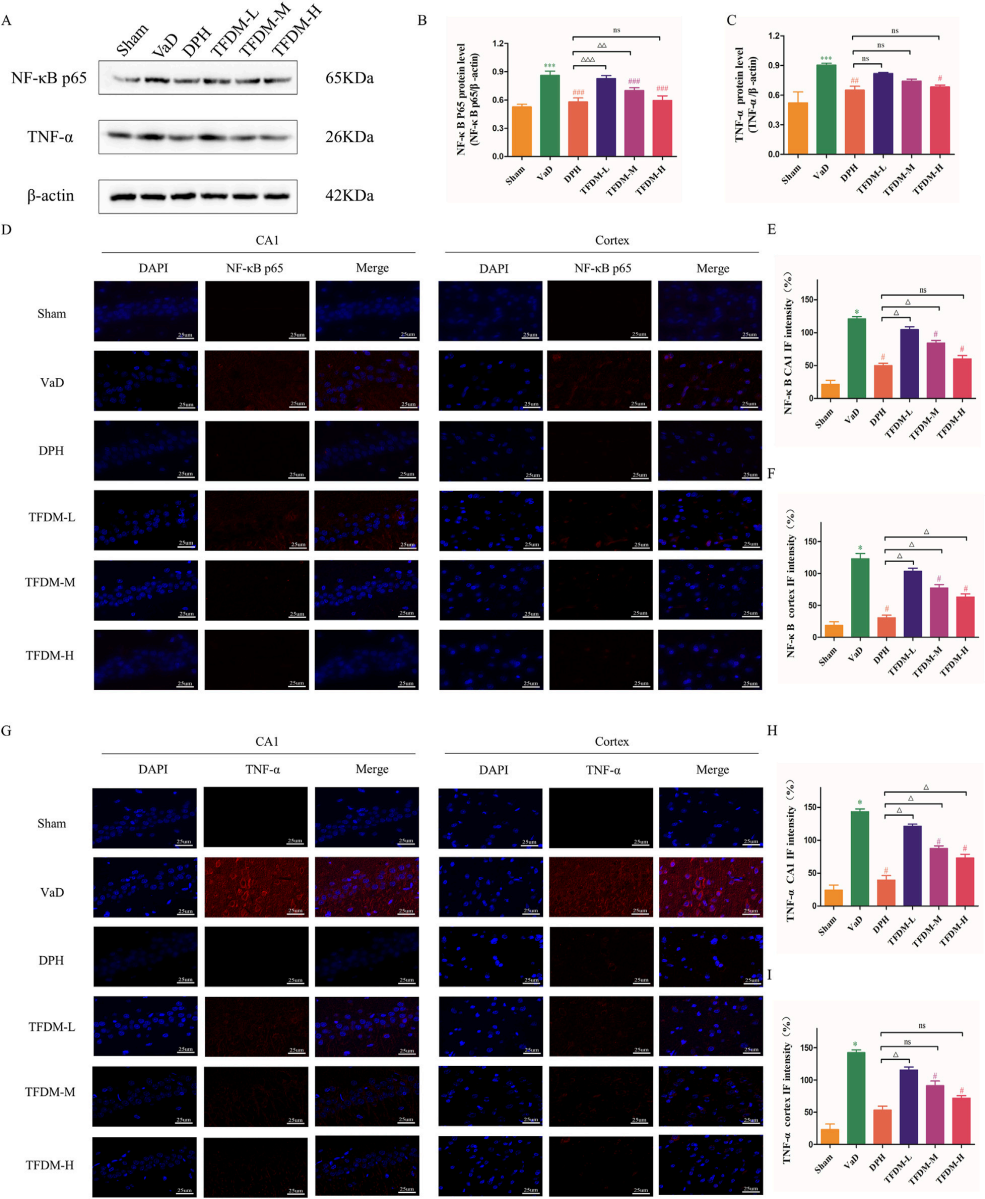
TFDM inhibited the TNF-α/NF-κB p65 signaling pathway in the brain tissue of VaD rats. **(A)** Representative image of Western blotting assays evaluating TNF-α and NF-κB p65 protein levels in rat brain tissue from each group. **(B,C)** Quantification results of the Western blot assay. **(D–F)** The immunofluorescence expression of NF-κB p65 (red) in the cortex and CA1 region of the hippocampus (25um). **(G–I)** The immunofluorescence expression of TNF-α(red) in the cortex and CA1 region of the hippocampus (25um). Data are expressed as mean ± SD (n = 5). The 100% reference represents the Sham group. ***P < 0.001, compared with the sham group. ###*P* < 0.001, ##*P* < 0.01, #*P* < 0.05, compared with the VaD group. ΔΔΔ*P* < 0.001, ΔΔ*P* < 0.01, Δ*P* < 0.05, compared with the DPH group.

As illustrated in Figure 6A, Western blot analysis was utilized to assess the proteins levels associated with the TNF-α/NF-κB p65 signaling pathway to elucidate the regulatory effects on neuroinflammation of TFDM. The results revealed significantly elevated NF-κB p65 and TNF-α protein levels in the VaD group compared to the Sham group (Figures 6B,C) (*P* < 0.001). These findings suggest that the TNF-α/NF-κB p65 pathway is activated following reduced cerebral blood flow and VaD development. Following drug intervention, TNF-α and NF-κB p65 protein levels decreased in the DPH, TFDM-M, and TFDM-H groups compared to the VaD group (*P* < 0.05 or *P* < 0.001). Meanwhile, Immunofluorescence staining showed that the fluorescence intensity of NF-κB p65 and TNF-α in the cortex and hippocampal CA1 region was increased in the VaD group compared to the Sham group (*P* < 0.001) (Figures 6D–I). However, the fluorescence intensity of NF-κB p65 and TNF-α in the cortex and hippocampal CA1 region was decreased in TFDM-M, TFDM-H groups, and DPH group compared to the VaD group (*P* < 0.001 or *P* < 0.01 or *P* < 0.05) (Figures 6D–I). However, no significant differences were observed between these treatment groups. These results indicate that TFDM treatment may exert its effects by inhibiting the TNF-α/NF-κB p65 signaling pathway, potentially mitigating neuroinflammation.

## 4 Discussion

Chronic cerebral hypoperfusion (CCH) is recognized as the hallmark of VaD (Rajeev et al., 2022). Cerebral ischemia and hypoxia-induced by hypoperfusion can engender pathological alterations, including oxidative stress, apoptosis, neuroinflammation, aberrant energy metabolism, and blood-brain barrier disruption. These changes can subsequently lead to neuronal deficits, either initiating or exacerbating the progression of VaD (Alexander et al., 2022; Butt et al., 2021).

This study obtained 169 key targets in TFDM-treated VaD through network topology. KEGG and GO enrichment analysis indicate that TFDM may prevent and treat VaD through reactive oxygen metabolism and the TNF signaling pathway. Next, the potential mechanism of TFDM in improving VaD was further validated through VaD rats. Our study represents the first investigation into the neuroprotective effects of TFDM against VaD-related neurodegeneration. TFDM treatment ameliorated cognitive dysfunction in VaD rats and significantly attenuated CCH-induced neuronal damage in the hippocampal CA1 region and cortex. TFDM treatment markedly reduced neuroinflammation, with the TNF-α/NF-κB p65 signaling pathway potentially playing a crucial role. Moreover, TFDM intervention improved blood-brain barrier permeability and increased angiogenesis in VaD rats. Collectively, TFDM showed a promising potential in the treatment of VaD, as might be mediated by the regulation of TNF-α/NF-κB p65 signaling pathway.

The 2VO model, a well-established animal model, was employed to investigate the effects of CCH on cognitive function and its underlying mechanisms (Yang et al., 2022). The MWM test was utilized to assess the impact of TFDM treatment on spatial learning and memory capabilities with 2VO rats. Consistent with Xiao’s findings (Xiao et al., 2023), the VaD group exhibited significantly longer escape latencies and swimming distances than the Sham group from day 1 to day 5, indicating that CCH induced a decline in spatial learning and memory abilities, ultimately resulting in cognitive impairment. On Day 6 of the probe trial, TFDM treatment significantly increasd the number of accesses to the platform and time in target quadrant. These results suggest that TFDM treatment substantially improves cognitive function in VaD rats.

The etiology of VaD is primarily attributed to chronic ischemia and hypoxia in brain tissue. The CA1 region of the hippocampus, which is most closely associated with memory and learning, is particularly vulnerable to ischemic and hypoxic conditions (Gao et al., 2022; Ma et al., 2024). To further elucidate the molecular mechanism by which TFDM ameliorates cognitive impairment in 2VO rats, this study focused on the hippocampal CA1 area and cortex. The experimental results revealed severe staining of neurons in the hippocampal CA1 region and cortical areas, demonstrating apparent pathological abnormalities consistent with previous studies. Nissl staining analysis confirmed a substantial reduction in neuronal loss and damage following TFDM treatment, corroborating the behavioral findings from the MWM test. The current research demonstrates that TFDM exerts a neuroprotective effect on VaD, significantly improving cognitive dysfunction and attenuating neuronal damage in VaD rats, thus presenting a potential preventive and therapeutic approach for VaD.

Neuroinflammation has been identified as a significant risk factor for CCH and neurodegenerative disorders, playing a crucial role in VaD (Chen et al., 2024). The interaction of neuroinflammation with various pathways throughout CCH development ultimately results in cognitive impairment (Zhao et al., 2021). Among these pathways, the activation of microglial cells regulates the progression of neuroinflammation (Wendimu and Hooks, 2022). Furthermore, inflammatory substances released by activated microglia, including interleukins (ILs) and TNF-α, exacerbate white matter diffusion damage, demyelination, and axonal loss, leading to cognitive function deterioration (Glass et al., 2010). NF-κB is considered a significant pro-inflammatory factor. Upon activation, NF-κB is translocated into the nucleus, phosphorylated, and stimulates the transcription of pro-inflammatory genes (Taniguchi and Karin, 2018). Edaravone dexborneol has been shown to inhibit the anti-inflammatory effect of NF-κB on microglial cells, thereby mitigating cognitive impairment in rats with vascular dementia (Li L. et al., 2023). Epimedium exhibits potential for preventing VaD by attenuating neuroinflammation and improving the devastation of BBB via the TNF signaling pathway (Xie et al., 2022). Dl-3-n-Butylphthalide can reduce neuroinflammation and enhance learning and memory in VaD rats by inhibiting the expression of TNF-α and NF-κB (Li Q. et al., 2023). These studies have demonstrated that the TNF-α/NF-κB pathway is an essential mediator in the inflammatory responses associated with VaD. Consistent with Kang’s findings (Kang et al., 2022), our study revealed that in VaD rats induced by 2VO, TNF-α, and NF-κB p65 protein levels were upregulated. However, TFDM treatment significantly reduced TNF-α and NF-κB p65 protein levels in 2VO rats. These findings suggest that TFDM may prevent 2VO-induced neuronal damage by blocking the TNF-α/NF-κB p65 signaling pathway, thus maintaining a stable and balanced inflammatory microenvironment.

Additionally, disruption of the BBB is a significant factor contributing to the pathological damage caused by VaD. The BBB, a highly specialized brain endothelium structure within the central nervous system, prevents large molecules and toxic chemicals from entering the brain’s parenchymal tissue from the peripheral circulation (Armulik et al., 2010). Under CCH conditions, cellular hypoxia-ischemia induces aberrant alterations in BBB endothelial cells, leading to BBB destruction and a considerable decrease in cerebral blood flow (Rajeev et al., 2022). Reduced cerebral blood flow also causes pericyte edema, microglial and astrocyte activation, basement membrane disruption, and tight junction protein breakdown, further exacerbating BBB damage. During CCH progression, this damage interacts with neuroinflammation to induce cognitive impairment (Lim et al., 2024). The primary manifestations of neuroinflammation include peripheral leukocyte infiltration, microglial activation, and the release of pro-inflammatory substances that further compromise the BBB. Increased BBB permeability allows immune cells and pathogens to enter the brain, intensifying neuroinflammation (Rajeev et al., 2022; Wang et al., 2023). BBB disruption is associated with tight junction protein downregulation and plays a crucial role in the development of neurodegenerative diseases (Sweeney et al., 2019). ZO-1, an essential tight junction protein, facilitates connections between transmembrane proteins and maintains tight junction integrity (Zhang et al., 2018). Matrix metalloproteinase-9 (MMP-9), a proteolytic enzyme, degrades tight junction proteins such as ZO-1, increasing BBB permeability (Che et al., 2023). Consistent with Wang’s research findings (Wang et al., 2023), our study revealed that in VaD rats induced by CCH, MMP-9 protein levels were upregulated while ZO-1 was downregulated. Conversely, TFDM treatment increased ZO-1 protein levels and decreased MMP-9 protein levels. These findings suggest that TFDM treatment protects the permeability of the BBB, indicating that protecting BBB permeability could be a valuable therapeutic target.

**FIGURE 7:**
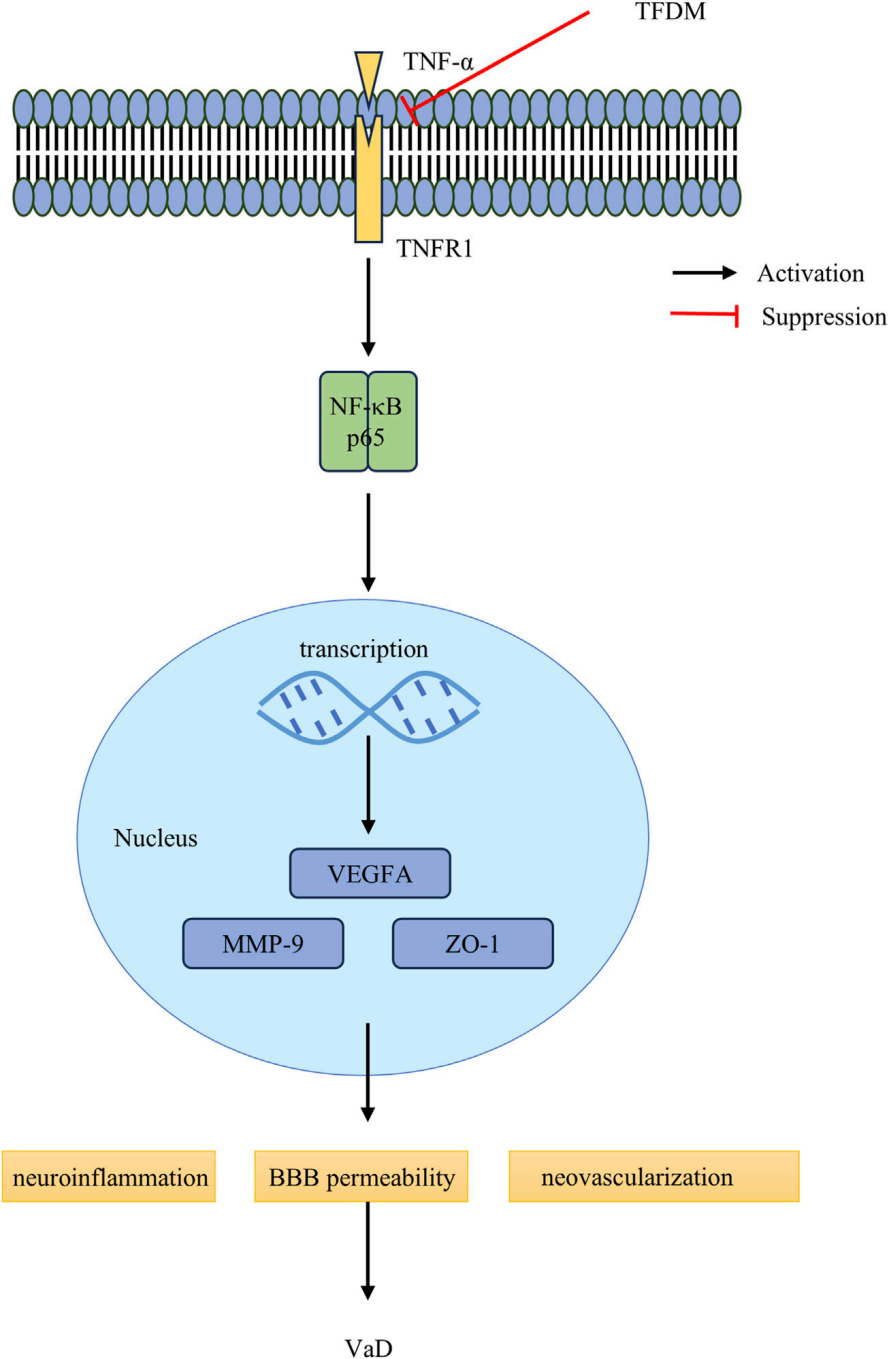
The schematic diagram of TFDM inhibits the TNF-α/NF-κB p65 signaling pathway and improves the blood-brain barrier to rescue VaD injuries. In the VaD rats, the binding of TNFR1 to TNF-α,which contributes to NF-κB p65, activation facilitates, facilitating the translocation of NF-κB p65 proteins into the nucleus for the regulation of target gene transcription. TFDM intervened by inhibiting the TNF-α and NF-κB p65, restraining the nuclear translocation of NF-κB p65, and ultimately repressing the excessive activation of the NF-κB p65 signaling pathway. Furthermore, TFDM Improves brain injuries by Protecting the Blood-brain Barrier and enhances angiogenesis, including Downregulation of MMP-9 protein levels and upregulation of ZO-1 protein levels. Meanwhile, TFDM protection from VaD injuries by upregulating the VEGFA protein levels, protecting BBB permeability.

VEGFA, an essential member of the VEGF family, is responsible for permeabilizing blood vessels, promoting cell migration, and stimulating endothelial cell growth (Rauniyar et al., 2023). VEGF enhances BBB permeability, a prerequisite for angiogenesis stimulation (Greenberg and Jin, 2013). Recent studies have linked VaD to altered VEGFA expression and protein levels in both the blood and brain, which may be involved in microvessel loss and BBB breakdown (Inoue et al., 2023; Pérez-Gutiérrez and Ferrara, 2023; Trares et al., 2022). VEGFA may exert a protective effect by preventing cognitive impairment (Yang et al., 2024). These findings imply that vascular dysfunction associated with cognitive impairment may be mitigated by reducing vascular inflammatory activation and reestablishing efficient angiogenesis (Tsartsalis et al., 2024). Our study has demonstrated that TFDM treatment can promote neovascularization by regulating VEGFA protein levels, thus enhancing the learning and memory abilities of VaD rats. The schematic diagram of TFDM improvement VaD injuries is shown in Figure 7.

This study has several limitations. Firstly, multiple upstream and downstream regulators are involved in the TNF-α/NF-κB p65 signaling pathway, which warrants further investigation in future research. Secondly, the intricate nature of BBB interactions and the modest sample size of examined variables leave many BBB mechanisms unidentified. Lastly, measuring cerebral blood flow could help confirm the potential BBB function suggested by this study.

## 5 Conclusion

In conclusion, our research revealed that TFDM treatment ameliorated cognitive impairment in VaD rats by inhibiting neuroinflammation, protecting BBB permeability, and promoting angiogenesis. The suppression of the TNF-α/NF-κB p65 signaling pathway could be associated with the neuroprotective action of TFDM. Furthermore, BBB modulation might represent a viable therapeutic target for VaD. While TFDM shows promise in mitigating VaD pathology, future studies should address the bioavailability of TFDM in humans and potential clinical formulations to improve cognitive impairment.

## Data Availability

The raw data supporting the conclusions of this article will be made available by the authors, without undue reservation.
